# Localizing Syntactic Composition with Left-Corner Recurrent Neural Network Grammars

**DOI:** 10.1162/nol_a_00118

**Published:** 2024-04-01

**Authors:** Yushi Sugimoto, Ryo Yoshida, Hyeonjeong Jeong, Masatoshi Koizumi, Jonathan R. Brennan, Yohei Oseki

**Affiliations:** Graduate School of Arts and Sciences, University of Tokyo, Tokyo, Japan; Graduate School of International Cultural Studies, Tohoku University, Sendai, Japan; Department of Linguistics, Graduate School of Arts and Letters, Tohoku University, Sendai, Japan; Department of Linguistics, University of Michigan, Ann Arbor, MI, USA

**Keywords:** fMRI, left-corner parsing, naturalistic reading, recurrent neural network grammar, surprisal, syntax

## Abstract

In computational neurolinguistics, it has been demonstrated that hierarchical models such as recurrent neural network grammars (RNNGs), which jointly generate word sequences and their syntactic structures via the syntactic composition, better explained human brain activity than sequential models such as long short-term memory networks (LSTMs). However, the vanilla RNNG has employed the top-down parsing strategy, which has been pointed out in the psycholinguistics literature as suboptimal especially for head-final/left-branching languages, and alternatively the left-corner parsing strategy has been proposed as the psychologically plausible parsing strategy. In this article, building on this line of inquiry, we investigate not only whether hierarchical models like RNNGs better explain human brain activity than sequential models like LSTMs, but also which parsing strategy is more neurobiologically plausible, by developing a novel fMRI corpus where participants read newspaper articles in a head-final/left-branching language, namely Japanese, through the naturalistic fMRI experiment. The results revealed that left-corner RNNGs outperformed both LSTMs and top-down RNNGs in the left inferior frontal and temporal-parietal regions, suggesting that there are certain brain regions that localize the syntactic composition with the left-corner parsing strategy.

## INTRODUCTION

Recent developments in computational linguistics and natural language processing have developed various kinds of computational models that can be employed to investigate neural computations in the human brain (e.g., Schrimpf et al., 2021), providing a new approach to the neurobiology of language (Hale et al., 2022). Specifically, computational models have played an important role to test linguistic theories against human brain activity, and the previous literature have examined whether natural languages are represented as hierarchical syntactic structures or linear word sequences (Chomsky, 1957; Everaert et al., 2015). For example, Frank et al. (2015) demonstrated that sequential models like recurrent neural networks (RNNs) successfully predict human electroencephalography (EEG) relative to context-free grammars (CFGs), suggesting that human language processing is insensitive to hierarchical syntactic structures. In contrast, the positive results of hierarchical models like CFGs and more expressive grammar formalisms like minimalist grammars and combinatory categorial grammars have also been confirmed against human EEG (Brennan & Hale, 2019) as well as functional magnetic resonance imaging (fMRI) (Brennan et al., 2016; Stanojević et al., 2023).

Moreover, the hybrid computational model of RNNs and CFGs has been proposed in the computational linguistics/natural language processing literature, namely recurrent neural network grammars (RNNGs; Dyer et al., 2016) which jointly generate word sequences and their syntactic structures via the syntactic composition. Interestingly, RNNGs outperformed sequential models like long short-term memory networks (LSTMs) in predicting not only syntactic dependencies (Kuncoro et al., 2018; Wilcox et al., 2019) and human eye movement (Wilcox et al., 2020; Yoshida et al., 2021), but also human brain activity like EEG (Hale et al., 2018) and fMRI (Brennan et al., 2020). These results indicate that RNNGs are the neurobiologically plausible computational model of human language processing.

However, the vanilla RNNG in Hale et al. (2018) and Brennan et al. (2020) has employed the top-down parsing strategy, which has been pointed out in the psycholinguistics literature as suboptimal especially for head-final/left-branching languages, and alternatively the left-corner parsing strategy has been proposed as the psychologically plausible parsing strategy (Abney & Johnson, 1991; Resnik, 1992). In addition, the recent result reported the positive results of the left-corner parsing strategy modeling self-paced reading and human eye movement (Oh et al., 2022).

In this article, building on this line of inquiry, we investigate not only whether hierarchical models like RNNGs better explain human brain activity than sequential models like LSTMs, but also which parsing strategy is more neurobiologically plausible. Specifically, there are two components in this paper. The first component is to construct a novel fMRI corpus named BCCWJ-fMRI where participants read newspaper articles selected from the Balanced Corpus of Contemporary Written Japanese (BCCWJ; Maekawa et al., 2014) through the naturalistic fMRI experiment. The second component is to evaluate computational models such as LSTMs, top-down RNNGs, and left-corner RNNGs against the novel fMRI corpus developed above. Importantly for the purpose here, given that Japanese is a head-final/left-branching language, this language should serve as an excellent testing ground to differentiate top-down and left-corner parsing strategies. To preview our results, we demonstrate that left-corner RNNGs outperform both LSTMs andtop-down RNNGs in the left inferior frontal and temporal-parietal regions, suggesting that there are certain brain regions that localize the syntactic composition with the left-corner parsing strategy.

## MATERIALS AND METHODS

### fMRI Corpus

In this subsection, we describe a novel fMRI corpus named BCCWJ-fMRI, that is, BCCWJ experimentally annotated with human fMRI.

#### Participants and stimuli

Forty-two Japanese native speakers were recruited (19 females and 23 males, range: 18–24 years old, mean age = 21.1, *SD* = 1.7). At the time of the experiment, all of them were undergraduate and graduate students at Tohoku University, which is located in the northern part of Japan. All participants were right handed and had normal or corrected-to-normal vision without any neurological deficits. For each participant, written informed consent was obtained prior to the experiment.

Stimuli for this experiment consisted of 20 newspaper articles from the BCCWJ (Maekawa et al., 2014). BCCWJ consists of 100 million words, which includes various texts such as books, newspapers, blogs, laws, and so forth. Like BCCWJ-EEG (Oseki & Asahara, 2020), the newspaper articles were all segmented into phrasal units instructed by the National Institute for Japanese Language and Linguistics. The 20 newspaper articles were divided into four blocks (A, B, C, D). Each block lasted for around 7 min excluding the first 20 s that the stimuli were not presented and 31 s for reading and answering the comprehension questions.

#### Procedure

During scanning, the stimuli were presented using rapid serial visual presentation (RSPVP) with PsychoPy (Peirce, 2007, 2009) where each segment was presented for 500 ms followed by a blank screen for 500 ms. Each participant read all blocks (A, B, C, D) in a randomized order. For each article, one yes–no comprehension question was given.

#### MRI acquisition and preprocessing

Scanning was conducted using the Philips Achieva 3.0T MRI scanner. During fMRI scanning, T2*-weighted MR signals were measured using a echo planar imaging pulse sequence (parameters: repetition time [TR] = 2,000 ms, echo time = 30 ms, flip angle = 80°, slice thickness = 4 mm, no slice gap, field of view = 192 mm, matrix = 64 × 64, and voxel size = 3 × 3 × 4). T1-weighted high-resolution anatomical images were also obtained (parameters: thickness = 1 mm, field of view = 256 mm, matrix = 368 × 368, repetition time = 1,100 ms, echo time = 5.1 ms) from each participant to use for preprocessing.

The obtained fMRI data were pre-processed using MATLAB (MathWorks, Natick, MA, USA) and Statistical Parametric Mapping (SPM12) software. The preprocessing included correction for head motion (realignment), slice timing correction, co-registration to theanatomical image, segmentation for normalization, spatial normalization using the Montreal Neurological Institute (MNI) template, and smoothing using a Gaussian filter with a full-width at a half-maximum (FWHM) of 8 mm.

### Computational Models

#### 5-gram models

5-gram models are a sequential model, which processes a word sequence without explicitly modeling its hierarchical structures. 5-gram models treat the context as a fixed window (Markov model), so it works as a weak sequential baseline for hierarchical models. We used 5-gram models (a fifth-order Markov language model with Keneser-Ney Smoothing) implemented with KenLM (Heafield, 2011).

#### Long short-term memory networks

LSTMs (Hochreiter & Schmidhuber, 1997) are a sequential model, which processes a word sequence without explicitly modeling its hierarchical structure. LSTMs can maintain the whole context as a single vector representation, so they work as a strong sequential baseline for hierarchical models. We used 2-layer LSTMs with 256 hidden and input dimensions. The implementation by Gulordava et al. (2018) was employed.

#### Recurrent neural network grammars

Recurrent neural network grammars (RNNGs) are a hierarchical model, which jointly models a word sequence and its syntactic structure. RNNGs rely on a stack LSTM to keep the previously processed partial parse and compress them into a single vector representation. At each step of processing, one of the following actions is selected:

- GEN: Generate a terminal symbol.
- NT: Open a nonterminal symbol.
- REDUCE: Close a nonterminal symbol that was opened by NT.

During a REDUCE action, the composition function based on the bidirectional LSTMs is executed; in both directions, constituents of the closed nonterminal are encoded and the single phrasal representation is calculated from the output of the forward and reverse LSTMs.

Two types of RNNGs were tested in our experiment; top-down RNNGs and left-corner RNNGs, namely, RNNGs that process the sentence and its syntactic structure in a top-down or left-corner fashion, respectively. We used RNNGs that had 2-layer stack LSTMs with 256 hidden and input dimensions. The implementation by Noji and Oseki (2021) was employed.

For inference of RNNGs, word-synchronous beam search (Stern et al., 2017) was employed. Word-synchronous beam search retains a collection of the most likely syntactic structures that are predicted given an observed partial sentence and marginalizes their probabilities to approximate the next word probability given the context. Although RNNGs can be employed in different beam sizes, we used the top-down RNNG with beam size *k* = 1,000 and the left-corner RNNG with beam size *k* = 400 for this study, based on Yoshida et al. (2021).

We utilized the computational models trained by Yoshida et al. (2021). Yoshida et al. (2021) trained these language models (LMs) on the National Institute for Japanese Language and Linguistics Parsed Corpus of Modern Japanese (2016), which comprises 67,018 sentences annotated with syntactic structures. The sequential LMs, the 5-gram model and LSTM, were trained with terminals only (i.e., word sequences), while hierarchical LMs, top-down RNNGs and left-corner RNNGs, were trained with terminals and their syntactic structures. See Yoshida et al. (2021) for the details of hyperparameter settings.

To quantify the quality of the models, the perplexity for each model was calculated. The models were computed for the texts that consist of 20 Japanese newspaper articles from BCCWJ. The perplexity for each model is as follows: 5-gram models (195.58), LSTMs (166.52), the top-down RNNG with beam size 1,000 (177.84), and the left-corner RNNG with beam size 400 (166.92). The full list of the perplexity for each LM, including different beam size RNNGs is summarized in the Table 1.

**Table 1.** Perplexities for all language models.

	**5-gram model**	
	**195.58219659288633**	
	**LSTM**	
	**166.5213055276006**	
**Beam size**	**RNNGs_LC**	**RNNGs_TD**
100	170.60928610079003	242.71035859949953
200	168.48339005024133	210.0192442957164
400	166.9281371024315	190.74082279178688
600	166.47254386281034	183.05484955898646
800	166.2157373706272	180.354934799703
1,000	165.99643995526114	177.8459006375216

*Note*. LSTM = long short-term memory.

### Evaluation Metrics

#### Surprisal

In order to test the output of LMs against fMRI data, surprisal was employed (Hale, 2001, 2016; Levy, 2008). Surprisal, an information-theoretic metric, logarithmically links probability estimation from the computational models with cognitive efforts from humans. Formally, surprisal is calculated as the negative log probability of the segment in its context.

$$-\log\;p\left(\text{segment}|\text{context}\right)$$

When the surprisal increases, there should be longer reading times or greater neural activities. In this study, we utilized the blood oxygen level-dependent (BOLD) signal as the measure of cognitive effort from humans.

#### Distance

In addition to surprisal, distance for RNNGs was employed in this study. This metric quantifies “syntactic work” where the number of parser actions (e.g., GEN, NT, REDUCE) is counted (Hale et al., 2018). Since RNNGs jointly model a word sequence and its syntactic structure, the word-synchronous beam search algorithm (Stern et al., 2017) is adopted to resolve the imbalance of the probability of the strings and the probability of the trees that RNNGs generate. This algorithm resolves this imbalance by considering “enough” potential parser actions. Distance is calculated by counting the number of these actions in the beam for each segment. Because this metric considers the number of actions in the beam, it is a more direct way of exploring the measure of cognitive effort of the syntactic processing in the brain.

Intuitively speaking, this metric is similar to the node count metric (e.g., Brennan et al., 2012, 2016), but not identical. These two metrics are similar in that they consider syntactic structures. The difference is that node count is applied to syntactic structures that are already constructed (i.e., a perfect oracle; cf. Brennan, 2016; Hale, 2014), whereas distance is counting the process and considering alternative structures that are potentially correct structures at the end of the sentence. Since this metric can only be employed for RNNGs, distance becomes relevant when RNNGs with different parsing strategies are compared in this study.

### Statistical Analyses

Before the statistical analysis, data from four participants were excluded due to an incomplete acquisition issue during the scanning in the MRI scanner (the scan stopped earlier than the designed time due to the experimenter’s error). Data from two participants were excluded due to the excessive head movement and data from two participants were excluded due to poor performance of the comprehension questions. Thus, data from 34 participants were used for data analysis.

#### Regions of interest analyses

Eight regions of interest (ROIs) in the left hemisphere were selected for this study based on previous work on the cognitive neuroscience of language literature (Bemis & Pylkkänen, 2011, 2013; Friederici, 2017; Hagoort, 2016; Matchin & Hickok, 2020; Zaccarella & Friederici, 2015). The ROIs chosen are the pars operularis (IFGoperc), the pars triangularis (IFGtriang), the pars orbitalis (IFGorb), the inferior parietal lobule (IPL), the angular gyrus (AG), the superior temporal gyrus (STG), the superior temporal pole (sATL), and the middle temporal pole (mATL). These regions were defined by automated anatomical labeling (AAL) atlas (Tzourio-Mazoyer et al., 2002). These regions are also motivated by the recent computational neurolinguistics literature (Brennan et al., 2016, 2020; Li & Hale, 2019; Lopopolo et al., 2017;  Lopopolo et al., 2021; Stanojević et al., 2021). In order to extract the BOLD signals for the ROI analyses, the parcellation was provided by AAL Atlas using nilearn (Version 0.9.2; Abraham et al., 2014; Nilearn, 2010; Pedregosa et al., 2012), a Python package for statistical analysis of neuroimaging data.

In this work, we used control predictors that are not our theoretical interests but yet reflect human language processing. Word rate (word_rate) is an indicator that assigns 1 to the offset of the segment that was presented in the screen for 500 ms and 0 elsewhere. This predictor tracks the rate at which the segment is presented during participants read segments, which covers the broad brain activities that have to do with language comprehension (cf. Brennan et al., 2012). Word length (word_length) was also used as a predictor for the baseline model, which counts the number of characters for each segment. Word frequency (word_freq) is a predictor for the log mean of the word frequencies for each segment. The value of sentence ID (sentid) is the number that was assigned to sentences in each block and the value of the sentence position (sentpos) indicates the number of the position of segments within a sentence for each article. Overall, we included 11 control predictors including six head movement parameters (dx, dy, dz, rx, ry, rz).

The predictors of our theoretical interests are the surprisal estimated from the 5-gram model and LSTM, the surprisal computed from the top-down RNNG (surp_RNNG_TD) and the left-corner RNNG (surp_RNNG_LC), and the distance computed from the top-down RNNG (dis_RNNG_TD) and the left-corner RNNG (dis_RNNG_LC). These predictors were transformed into estimated BOLD signals via a canonical hemodynamic response function (HRF) in order. (i) We created segment-by-segment time series for the values of surprisal computed from the 5-gram model, LSTM, and RNNGs, and time series for the values of distance estimated from RNNGs. (ii) These values as well as the values from control predictors (word_rate, word_length, word_freq, sentid, and sentpos) were convolved with the HRF using nilearn (more specifically, using the function compute_regressor). The head movement parameters were excluded from this computation. (iii) The convolved values from the 5-gram model, LSTM, and RNNGs were orthogonalized against word_rate to isolate each predictor’s effect from the broad language processing effects. (iv) compute_regressor was done with re-sampling the values to 0.5 Hz to match the time series of the fMRI data (TR = 2.0). After executing compute_regressor, the output was concatenated with the fMRI time series from 34 individuals in the eight ROIs that are extracted using AAL Atlas via nilearn.

In Table 2, the Pearson correlation matrix between predictors excluding six head movement parameters is shown.

**Table 2.** Correlations among predictors (Pearson’s *r*).

	**word rate**	**word length**	**word freq**	**sentid**	**sentpos**	**Surprisal**				**Distance**	
						**5-gram**	**LSTM**	**RNNG_TD**	**RNNG_LC**	**RNNG_TD**	**RNNG_LC**
word rate	1.00										
word length	0.84	1.00									
word freq	0.996	0.83	1.00								
sentid	0.68	0.67	0.69	1.00							
sentpos	0.64	0.49	0.65	0.40	1.00						
5-gram	<0.01	0.49	−0.015	0.14	−0.13	1.00					
LSTM	<0.01	0.48	−0.017	0.14	−0.14	0.98	1.00				
surp_RNNG_TD	<0.01	0.48	−0.017	0.14	−0.13	0.98	0.99	1.00			
surp_RNNG_LC	<0.01	0.48	−0.02	0.15	−0.14	0.98	0.99	0.99	1.00		
dis_RNNG_TD	<0.01	0.39	0.018	0.13	−0.034	0.58	0.53	0.54	0.54	1.00	
dis_RNNG_LC	<0.01	0.33	0.015	0.15	0.13	0.48	0.43	0.43	0.44	0.84	1.00

*Note*. LSTM = long short-term memory.

Among predictors, word rate is highly correlated with word frequency (*r*(word rate, word freq) = 0.996) as well as word length (*r*(word rate, word freq) = 0.84). Word frequency and word length are also highly correlated (*r*(word freq, word length) = 0.83). Sentence ID is relatively correlated with word rate (*r*(word rate, sentid) = 0.68), word length (*r*(word length, sentid) = 0.67), and word frequency (*r*(word freq, sentid) = 0.69). The similar pattern can be seen for sentence position as well. In terms of predictors of our interests, 5-gram is highly correlated with LSTM and surp_RNNGs (*r*(5-gram, LSTM) = 0.98, *r*(5-gram, surp_RNNG_TD) = 0.98, and *r*(5-gram, surp_RNNG_LC) = 0.98). LSTM, and two surp_RNNGs are also highly correlated with each other (*r*(LSTM, surp_RNNG_TD) = 0.99, *r*(LSTM, surp_RNNG_LC) = 0.99, and *r*(surp_RNNG_TD, surp_RNNG_LC) = 0.99). The two predictors for distance are also relatively correlated (*r*(dis_RNNG_TD, dis_RNNG_LC) = 0.84), while these two predictors do not have a high correlation with the predictors such as 5-gram and LSTM (e.g., *r*(LSTM, dis_RNNG_LC) = 0.43).

Before analyzing data on R (Bates & Sarkar, 2006), we removed the first 20 s of the data for each block and all the predictors were standardized. The outliers were also removed from the values for each ROI. The baseline model was created using the function lmer from the lme4 package in R. For fixed effects, we included word rate, word length, word frequency, sentence ID, sentence position, and six head movement parameters. A random intercept by participant was also included. The baseline model was defined below using the Wilkinson-Rogers notation.

$$\text{ROI}∼\text{word\_rate}+\text{word\_length}+\text{word\_freq}+\text{sentid}+\text{sentpos}+dx+dy+dz+rx+ry+rz+\left(1|\text{subject\_number}\right)$$

Then we added the predictors in the following order; 5-gram, LSTM, surp_RNNG_TD, and surp_RNNG_LC. This order reflects the richness of the architectures, the hierarchical information, and the model performance shown in Yoshida et al. (2021). Model comparisons were done by the function anova(). After applying this function, the statistical significance was corrected for each *p* value by Bonferroni correction (*α* = 0.05/8 = 0.00625). Model comparison was also done with a model that includes control predictors, 5-gram, and LSTM, and a model that includes surp_RNNG_LC as well as the control predictors, and 5-gram, and LSTM to test whether surp_RNNG_LC has above-and-beyond effect for LSTM. We also constructed a model that includes control predictors, 5-gram, LSTM, surp_RNNG_LC and a model that includes surp_RNNG_TD as well as control predictors, 5-gram, LSTM, surp_RNNG_LC for model comparison to test whether the top-down RNNG has above-and-beyond effects for the left-corner RNNG.

Regarding distance, we constructed a regression model that includes the control predictors, 5-gram, and LSTM. Then we only added dis_RNNG_TD, and applied anova() to the model without dis_RNNG_TD and the model that includes dis_RNNG_TD. Then we added dis_RNNG_LC to the model to test whether the left-corner RNNG has above-and-beyond effects for the top-down RNNG. Model comparison was also done with a model that includes the control predictors, 5-gram, and LSTM, and a model that includes dis_RNNG_LC as well as the control predictors, 5-gram, and LSTM to test whether dis_RNNG_LC has above-and-beyond effect for LSTM. We also tested dis_RNNG_TD whether the top-down RNNG has above-and-beyond effects for the left-corner RNNG in the same way. The following list summarizes what this study tested in the ROI analyses. The boldface text indicates what we tested in this article.

1. **baseline model < n-gram < LSTM < surp_RNNG_TD < surp_RNNG_LC**
2. baseline model < n-gram < **LSTM < surp_RNNG_LC < surp_RNNG_TD**
3. baseline model < n-gram < **LSTM < dis_RNNG_TD < dis_RNNG_LC**
4. baseline model < n-gram < **LSTM < dis_RNNG_LC < dis_RNNG_TD**

#### Whole brain analyses

In addition to the ROI analyses, we also did an exploratory analysis independently. This analysis confirms the regions that are activated with respect to each predictor. Using nilearn package, the design matrices were created for the first-level general linear model. All predictors were included except for head movement parameters. The participant coefficient map was saved for the second-level analysis.

For the second-level analysis, one-sample *t* tests were performed. The threshold maps were *z*-valued and the threshold was defined as follows; false discovery rate was *α* = 0.05 and a threshold of the cluster size was 100 voxels. For the masking, Yeo et al.’s (2011) cortical mask was used and a FWHM Gaussian smoothing (8 mm) was applied. AtlasReader (Notter et al., 2019) was used for identifying the regions of peaks for each cluster size.

## RESULTS

### Behavioral Results

The mean number of correct responses across participants for the comprehension questions was 13.6 (*SD* = 3.6) out of 20 (68%).

### ROI Analyses

Table 3 shows the results of the model comparisons of 5-gram, LSTM, surp_RNNG_TD, and surp_RNNG_LC. These comparisons were done by sequentially adding terms of theoretical interests. We found no statistically significant effects across ROIs for both 5-gram and LSTM models. Furthermore, there are no statistically significant effects by just adding surp_RNNG_TD across ROIs. However, when surp_RNNG_LC was added and compared with the model without it, all ROIs except for mATL showed statistically significant effects even after corrected for multiple comparisons.

**Table 3.** Results of the model comparisons for 5-gram, LSTM, surp_RNNG_TD, and surp_RNNG_LC.

**ROIs**	**Model comparisons**	**LogLik**	***χ* ^2^**	***p***
IFGoperc	baseline < 5-gram	−9092.3	6.1327	0.17
	5-gram < LSTM	−9091.9	0.7985	0.372
	LSTM < RNNG_TD	−9090.8	2.2179	0.136
	RNNG_TD < RNNG_LC	−9072.5	36.622	<0.001*
IFGtriang	baseline < 5-gram	−11061	0.8954	0.344
	5-gram < LSTM	−11060	2.708	0.0998
	LSTM < RNNG_TD	−11060	0.3085	0.578
	RNNG_TD < RNNG_LC	−11041	37.239	<0.001*
IFGorb	baseline < 5-gram	−17918	0.1266	0.721
	5-gram < LSTM	−17918	0.4371	0.508
	LSTM < RNNG_TD	−17918	0.0008	0.977
	RNNG_TD < RNNG_LC	−17913	9.2683	0.002*
IPL	baseline < 5-gram	−12705	4.6624	0.03
	5-gram < LSTM	−12704	1.8846	0.169
	LSTM < RNNG_TD	−12702	5.9362	0.051
	RNNG_TD < RNNG_LC	−12667	70.28	<0.001*
AG	baseline < 5-gram	−13413	5.4511	0.019
	5-gram < LSTM	−13412	2.0618	0.151
	LSTM < RNNG_TD	−13410	3.7982	0.051
	RNNG_TD < RNNG_LC	−13390	41.065	<0.001*
STG	baseline < 5-gram	−13841	1.6733	0.195
	5-gram < LSTM	−13839	2.8784	0.089
	LSTM < RNNG_TD	−13837	4.0574	0.043
	RNNG_TD < RNNG_LC	−13822	31.524	<0.001*
sATL	baseline < 5-gram	−19064	3.2966	0.069
	5-gram < LSTM	−19064	0.0072	0.932
	LSTM < RNNG_TD	−19062	2.7917	0.094
	RNNG_TD < RNNG_LC	−19057	10.01	0.002*
mATL	baseline < 5-gram	−23917	5.2513	0.021
	5-gram < LSTM	−23917	0.0261	0.871
	LSTM < RNNG_TD	−23917	0.583	0.445
	RNNG_TD < RNNG_LC	−23916	1.5699	0.21

*Note*. ROI = region of interest, IFG = inferior front gyrus pars opercularis, IFGtriang = IFG pars triangularis, IFGorb = IFG pars orbitalis, IPL = inferior parietal lobule, AG = angular gyrus, STG = superior temporal gyrus, sATL = superior temporal pole, mATL = middle temporal pole. Bonferroni correction (*α* = 0.05/8 = 0.00625) was applied.

* Indicates improvement in model fit, using Bonferroni correction.

As Table 4 shows, we also tested whether surp_RNNG_LC has the above-and-beyond effects for LSTM. The results confirmed such effects in IFGoperc, IFGtriang, IPL, AG, and STG.

**Table 4.** Results of the model comparisons for testing whether either surp_RNNG_TD or surp_RNNG_LC improves the model fit to the fMRI data against LSTM (LSTM < {surp_RNNG_TD, surp_RNNG_LC}).

**ROIs**	**surp_RNNG_TD**			**surp_RNNG_LC**		
	**LogLik**	***χ* ^2^**	***p***	**LogLik**	***χ* ^2^**	***p***
IFGoperc	−9090.8	2.2179	0.136	−9085.2	13.33	<0.001*
IFGtriang	−11060	0.3085	0.578	−11050	18.427	<0.001*
IFGorb	−17918	8e−04	0.977	−17915	5.3059	0.021
IPL	−12702	4.0516	0.044	−12691	25.851	<0.001*
AG	−13410	3.7982	0.051	−13406	13.17	<0.001*
STG	−13837	4.0574	0.043	−13835	8.8692	0.0029*
sATL	−19062	2.7917	0.094	−19063	1.7879	0.181
mATL	−23917	0.583	0.445	−23917	0.2148	0.643

*Note*. Bonferroni correction (*α* = 0.05/8 = 0.00625) was applied.

* Indicates improvement in model fit, using Bonferroni correction.

The next statistical analysis summarized in Table 5 shows that surp_RNNG_TD better fits to IFGoperc, IFGtriang, IPL, AG, STG, and sATL, compared to surp_RNNG_LC.

**Table 5.** Results of the model comparison for testing whether surp_RNNG_TD has above-and-beyond effects for surp_RNNG_LC (surp_RNNG_LC < surp_RNNG_TD).

**ROIs**	**LogLik**	***χ* ^2^**	***p***
IFGoperc	−9072.5	25.51	<0.001*
IFGtriang	−11041	19.12	<0.001*
IFGorb	−17913	3.9633	0.0465
IPL	−12667	48.48	<0.001*
AG	−13390	31.693	<0.001*
STG	−13822	26.712	<0.001*
sATL	−19057	11.014	<0.001*
mATL	−23916	1.938	0.1639

*Note*. Bonferroni correction (*α* = 0.05/8 = 0.00625) was applied.

* Indicates improvement in model fit, using Bonferroni correction.

Regarding dis_RNNG_TD and dis_RNNG_LC, the results are summarized in Table 6. The results show that both dis_RNNG_TD and dis_RNNG_LC have statistically significant effects in several ROIs against LSTM; IFGoperc, IFGtriang, IPL, AG, and sATL for dis_RNNG_TD; and IFGoperc, IFGtriang, IFGorb, IPL, AG, STG, and sATL for dis_RNNG_LC respectively.

**Table 6.** Results of the model comparisons for testing whether either dis_RNNG_TD or dis_RNNG_LC improves the model fit to the fMRI data against LSTM (LSTM < {dis_RNNG_TD, dis_RNNG_LC}).

**ROIs**	**dis_RNNG_TD**			**dis_RNNG_LC**		
	**LogLik**	***χ* ^2^**	***p***	**LogLik**	***χ* ^2^**	***p***
IFGoperc	−9082.1	19.688	<0.001*	−9062.0	59.778	<0.001*
IFGtriang	−11055	8.7038	0.0031*	−11039	42.006	<0.001*
IFGorb	−17915	5.0968	0.023	−17907	21.882	<0.001*
IPL	−12695	17.437	<0.001*	−12682	44.454	<0.001*
AG	−13402	19.663	<0.001*	−13397	29.849	<0.001*
STG	−13836	6.948	0.008391	−13824	30.705	<0.001*
sATL	−19051	25.276	<0.001*	−19051	25.276	<0.001*
mATL	−23916	2.7588	0.096	−23915	4.3622	0.036

*Note*. Bonferroni correction (*α* = 0.05/8 = 0.00625) was applied.

* Indicates improvement in model fit, using Bonferroni correction.

Table 7 shows the results for testing whether dis_RNNG_LC better explains the fMRI data than dis_RNNG_TD. The results showed statistically significant effects in IFGoperc, IFGtriang, IFGorb, IPL, AG, and STG. On the other hand, there were no statistically significant effects in any ROIs when we tested whether dis_RNNG_TD better fits to the fMRI data, compared to dis_RNNG_LC (Table 8).

**Table 7.** Results of the model comparison for testing whether dis_RNNG_LC has above-and-beyond effects for dis_RNNG_TD (dis_RNNG_TD < dis_RNNG_LC).

**ROIs**	**LogLik**	***χ* ^2^**	***p***
IFGoperc	−9060.4	43.331	<0.001*
IFGtriang	−11035	40.385	<0.001*
IFGorb	−17905	19.752	<0.001*
IPL	−12681	28.113	<0.001*
AG	−13397	10.587	0.0011*
STG	−13822	28.142	<0.001*
sATL	−19051	0.099	0.753
mATL	−23915	1.6405	0.2003

*Note*. Bonferroni correction (*α* = 0.05/8 = 0.00625) was applied.

* Indicates improvement in model fit, using Bonferroni correction.

**Table 8.** Results of the model comparison for testing whether dis_RNNG_TD has above-and-beyond effects for dis_RNNG_LC (dis_RNNG_LC < dis_RNNG_TD).

**ROIs**	**LogLik**	***χ* ^2^**	***p***
IFGoperc	−9060.4	3.2412	0.0718
IFGtriang	−11035	7.0826	0.0077
IFGorb	−17905	2.9665	0.085
IPL	−12681	1.0961	0.295
AG	−13397	0.4008	0.526
STG	−13822	4.385	0.036
sATL	−19051	0.099	0.753
mATL	−23915	0.0371	0.847

*Note*. Bonferroni correction (*α* = 0.05/8 = 0.00625) was applied.

Table 9 summarizes the results of ROI analyses in this study.

**Table 9.** The summary of the main results from ROI analyses.

**Model comparison**	**IFGoperc**	**IFGtriang**	**IFGorb**	**IPL**	**AG**	**STG**	**sATL**	**mATL**
LSTM < surp_RNNG_LC	<0.001	<0.001		<0.001	<0.001	0.0029		
LSTM < surp_RNNG_TD								
LSTM < dis_RNNG_LC	<0.001	<0.001	<0.001	<0.001	<0.001	<0.001	<0.001	
LSTM < dis_RNNG_TD	<0.001	0.003		<0.001	<0.001		<0.001	

surp_RNNG_TD < surp_RNNG_LC	<0.001	<0.001	<0.001	<0.001	<0.001		<0.001	
surp_RNNG_LC < surp_RNNG_TD	<0.001	<0.001		<0.001	<0.001	<0.001	<0.001	

dis_RNNG_TD < dis_RNNG_LC	<0.001	<0.001	<0.001	<0.001	<0.001	<0.001		
dis_RNNG_LC < dis_RNNG_TD								

*Note*. *p* value was corrected by Bonferroni correction (*α* = 0.05/8 = 0.00625) for each model comparison.

A reviewer raised the question whether the beam size differences for RNNGs make different results. In order to answer this question, we did model comparison analyses where a regression model that includes the control predictors as well as 5-gram and LSTM and a model that includes one RNNG as well as the control predictors, 5-gram, and LSTM were tested via anova() using (i) different beam sizes (*k* = 100, 200, 400, 600, 800, 1,000), (ii) different parsing strategies (top-down or left-corner), and (iii) different complexity metrics (*surprisal* and *distance*) of RNNGs. The details of the results are summarized in the Supporting Information, available at https://doi.org/10.1162/nol_a_00118. Overall, regardless of the beam size differences or complexity metrics, the left-corner RNNGs improve the model fit to the fMRI data, compared to LSTM. On the other hand, the surprisal estimated from top-down RNNGs only improve the model fit to the fMRI data when the beam size is small (*k* = 100, 200). The distance computed from top-down RNNGs improves the model fit to the fMRI data regardless of the beam size differences.

### Whole Brain Analyses

**Figure 1.**
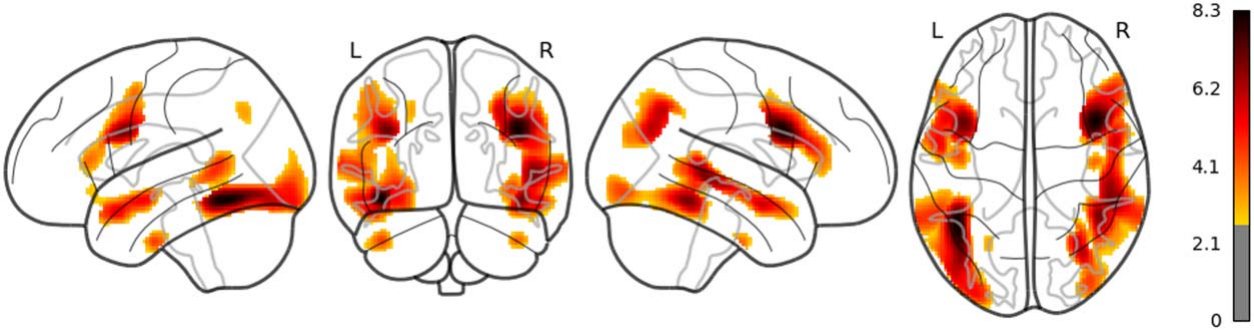
The result of whole brain analysis of word_rate.

**Figure 2.**
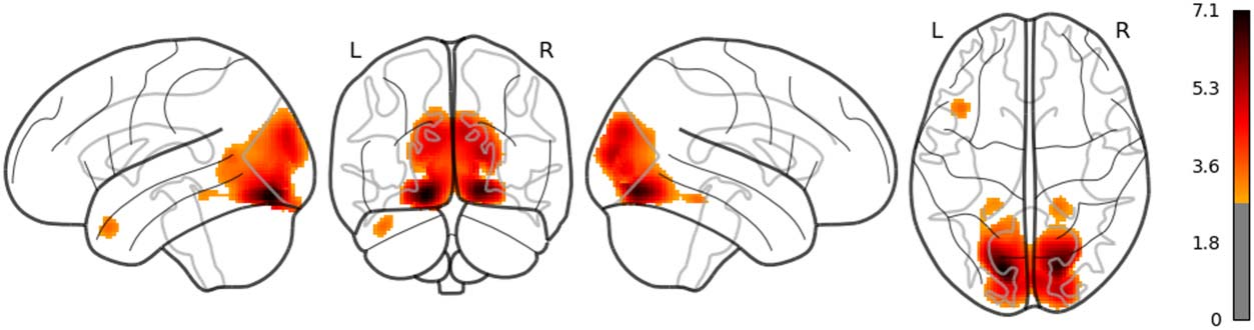
The result of whole brain analysis of word_length.

**Figure 3.**
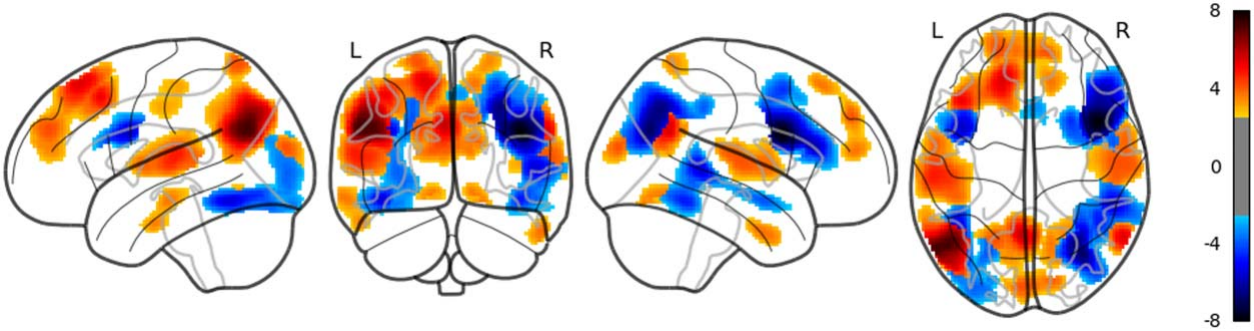
The result of whole brain analysis of word_freq.

**Figure 4.**
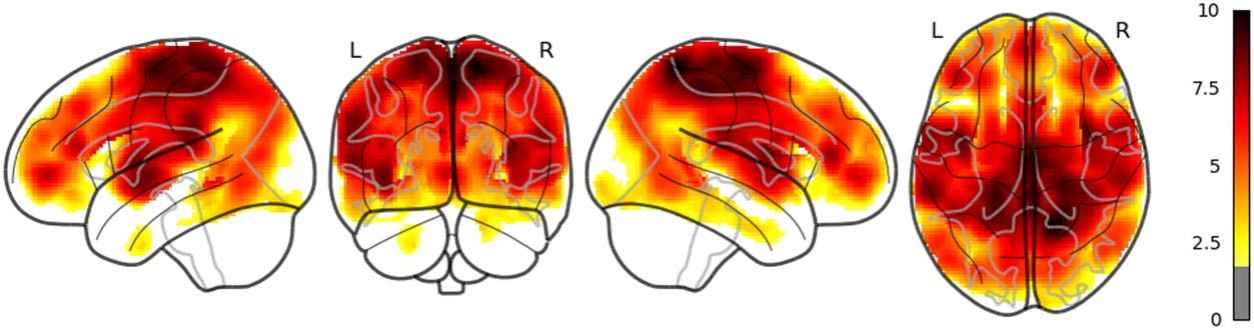
The result of whole brain analysis of sentid.

**Figure 5.**
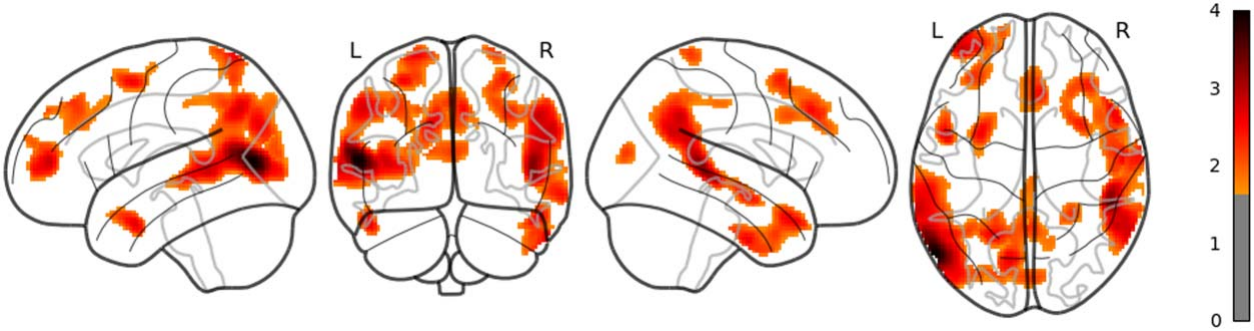
The result of whole brain analysis of sentpos (uncorrected).

For the control predictors, the following results were obtained from the whole brain analysis (Table 10 and Figures 1–5).

**Table 10.** The coefficient results of GLM for word rate, word length, word frequency, sentence ID and sentence position.

**Predictors**	**MNI coordinates**			**peak_stat (z)**	**Cluster size (mm^3^)**	**Region (AAL)**
	**peak_x**	**peak_y**	**peak_z**			
word_rate	44	−46	−16	6.86467	26,728	Fusiform_R
	−42	−54	−14	8.28593	23,624	Fusiform_L
	42	10	28	8.17836	14,200	Frontal_Inf_Oper_R
	−38	8	26	7.02740	11,608	Frontal_Inf_Oper_L
	32	−68	30	6.07489	7,256	Occipital_Mid_R
	−48	12	−22	5.53063	6,552	Temporal_Pole_Sup_L
	−42	−10	−40	4.51107	1,424	Temporal_Inf_L
	−24	−64	40	3.27936	544	Occipital_Mid_L
	40	−16	−40	3.98876	504	no_label
word_length	−16	−80	−12	7.13176	60,352	Lingual_L
	−40	18	−32	3.42982	840	Temporal_Pole_Mid_L
word_freq	−16	36	56	5.40559	32,456	Frontal_Sup_2_L
	42	10	26	−7.97979	23,384	Frontal_Inf_Oper_R
	−42	−56	−16	−5.50034	22,016	Fusiform_L
	−56	−68	28	7.28381	19,464	no_label
	−58	−24	12	4.78592	19,384	Temporal_Sup_L
	32	−70	28	−6.83518	17,960	Occipital_Mid_R
	52	−40	4	−5.47845	17,320	Temporal_Mid_R
	−4	−58	30	5.68754	14,352	Precuneus_L
	54	−16	10	4.09421	13,800	Rolandic_Oper_R
	−40	6	26	−5.70568	6,152	Frontal_Inf_Oper_L
	−2	−90	18	4.10801	5,640	Cuneus_L
	58	−60	28	5.47120	4,312	Angular_R
	−64	−20	−14	3.62544	4,096	Temporal_Mid_L
	−48	−24	54	3.15662	3,256	Postcentral_L
	−20	−60	68	4.09517	2,840	Parietal_Sup_L
	56	−2	−34	3.69464	1,976	Temporal_Inf_R
	14	−64	−12	3.62328	1,672	Lingual_R
	24	−58	70	3.39034	1,600	Parietal_Sup_R
	−12	−66	−12	3.66282	1,536	Cerebelum_6_L
	6	18	48	−3.28625	1,184	Supp_Motor_Area_R
sentid	16	−54	66	10.05250	694,224	Parietal_Sup_R
	−22	−2	−44	3.06418	1,512	Fusiform_L
	−32	−36	−28	2.22755	56	Cerebelum_4_5_L
sentpos (uncorrected)	−56	−70	8	3.98667	18,664	Temporal_Mid_L
	50	−38	4	3.26623	12,440	Temporal_Mid_R
	−10	−72	32	2.61551	3,776	Precuneus_L
	−40	54	8	2.80792	3,144	Frontal_Mid_2_L
	58	−12	−38	2.59958	3,120	Temporal_Inf_R
	−22	−60	72	2.59144	1,640	Parietal_Sup_L
	8	28	40	2.49773	1,368	Frontal_Sup_Medial_R
	36	32	38	2.38693	1,104	Frontal_Mid_2_R
	−28	4	58	2.57084	1,072	Frontal_Mid_2_L
	−50	0	−30	2.66369	1,016	Temporal_Mid_L
	32	8	58	2.40335	832	Frontal_Mid_2_R
	−34	38	44	2.23455	768	Frontal_Mid_2_L
	2	−84	14	2.19358	640	Calcarine_L
	26	−46	74	2.21470	240	Postcentral_R
	44	14	38	2.07152	176	Frontal_Inf_Oper_R
	−20	−82	18	2.08603	112	Occipital_Mid_L
	−16	62	20	2.00798	32	Frontal_Sup_2_L

*Note*. Thresholded with a false discovery rate = 0.05 and a cluster threshold of 100 voxels. The regions were identified by using AtlasReader (Notter et al., 2019). MNI = Montreal Neurological Institute, AAL = automated anatomical labeling.

The main results are reported as follows: Word rate (Figure 1) was associated with the activation in the bilateral fusiform gyri, bilateral middle occipital lobes, and the bilateral inferior frontal gyri (opercular part). Word length (Figure 2) was associated with the activation in the left lingual gyrus and the left middle temporal pole. Part of these results indicate that word rate and word length predictors are involved in the activities in the visual processing and the visual word form area.

**Figure 6.**
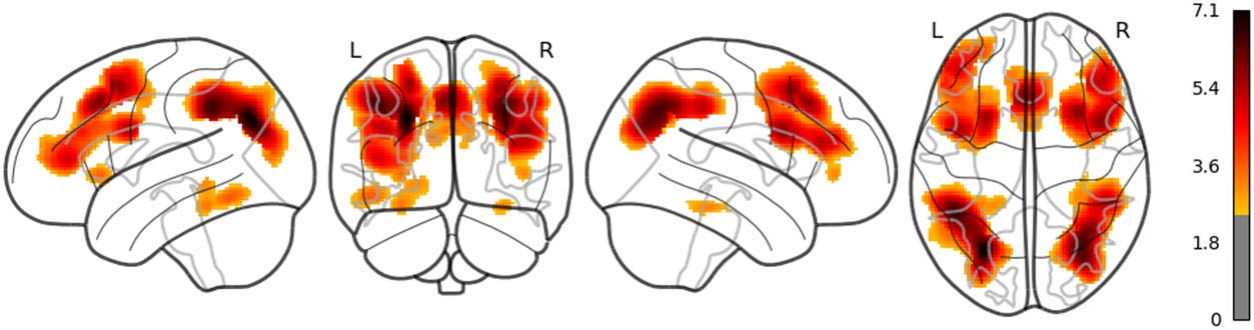
The result of whole brain analysis of 5-gram.

**Figure 7.**
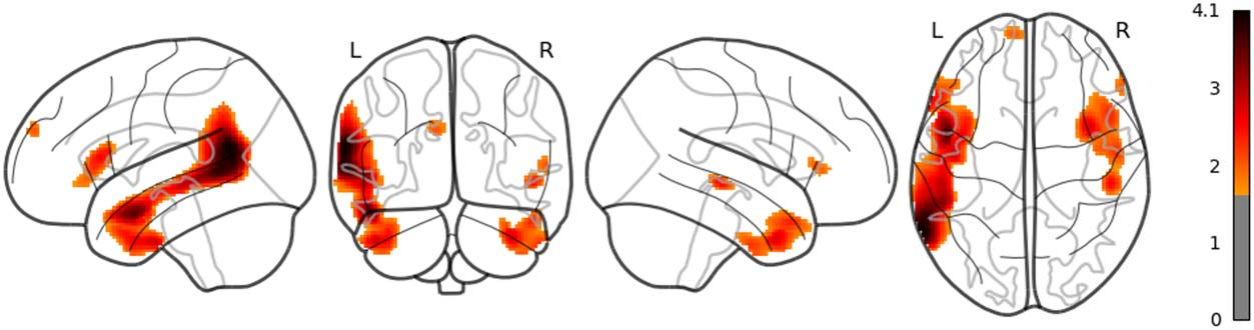
The result of whole brain analysis of LSTM (uncorrected).

**Figure 8.**
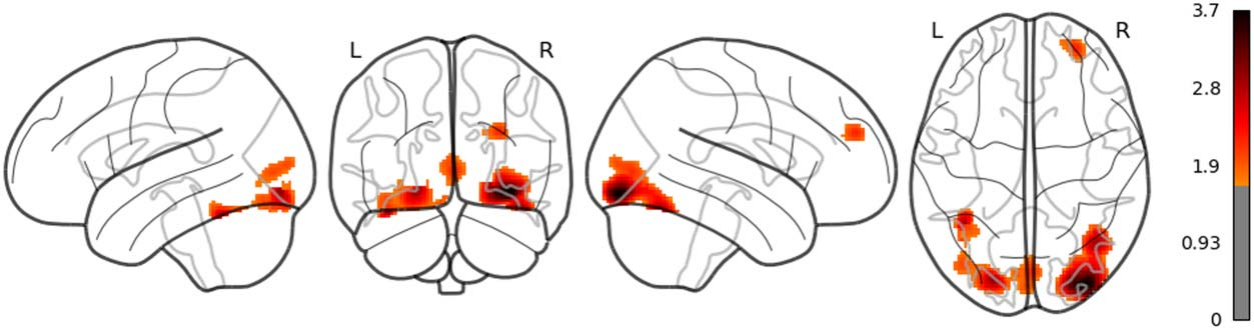
The result of whole brain analysis of surp_RNNG_TD (uncorrected).

**Figure 9.**
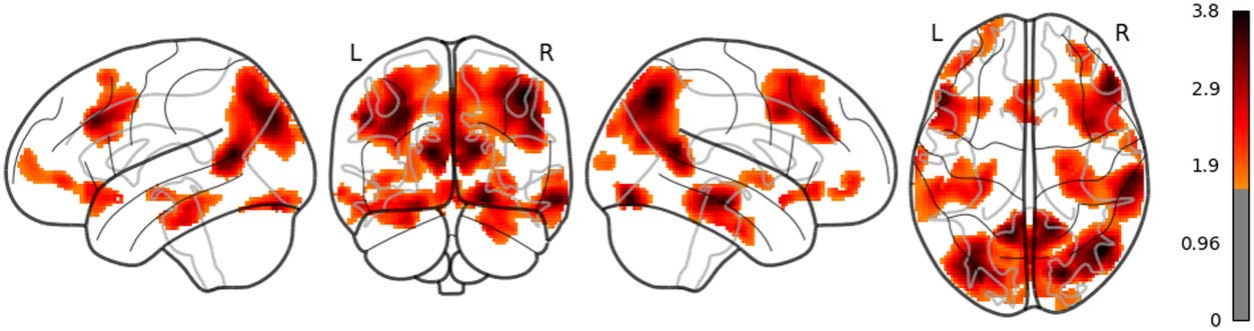
The result of whole brain analysis of surp_RNNG_LC (uncorrected).

**Figure 10.**
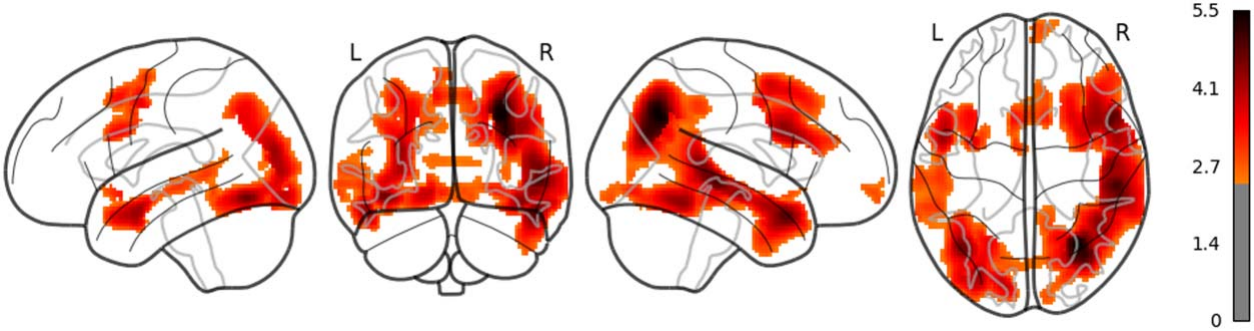
The result of whole brain analysis of dis_RNNG_TD.

**Figure 11.**
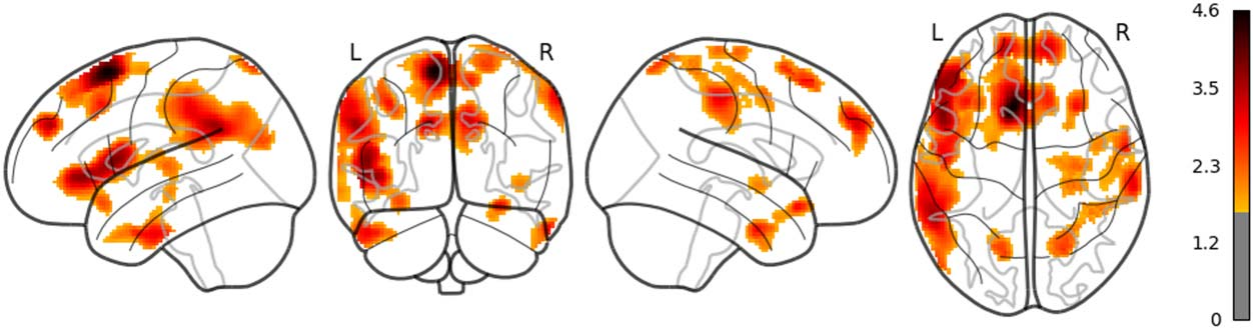
The result of whole brain analysis of dis_RNNG_LC (uncorrected).

Our main interests are the results of the whole brain analysis for LSTM, the top-down RNNG, and the left-corner RNNG, which are summarized in Table 11 (see also Figures 6–11).

**Table 11.** The coefficient results of GLM for the 5-gram, LSTM, surp_RNNG_TD, surp_RNNG_LC, dis_RNNG_TD, and dis_RNNG_LC.

**Predictors**	**MNI coordinates**			**peak_stat (z)**	**Cluster size (mm^3^)**	**Region (AAL)**
	**peak_x**	**peak_y**	**peak_z**			
5-gram	30	16	52	5.72940	31,824	Frontal_Mid_2_R
	−26	12	54	5.63596	30,648	Frontal_Mid_2_L
	−26	−68	34	7.14751	29,608	Occipital_Mid_L
	32	−66	40	6.49177	28,344	Occipital_Sup_R
	−4	24	44	5.85200	14,128	Frontal_Sup_Medial_L
	−48	−56	−14	3.55410	3,504	Temporal_Inf_L
	−30	−42	−18	3.26173	2,432	Fusiform_L
	32	−36	−20	3.15951	1,352	Fusiform_R
	−30	24	0	3.42302	1,096	Insula_L
LSTM (uncorrected)	−64	−56	16	4.05139	23,904	no_label
	38	6	−38	2.74617	2,752	Temporal_Pole_Mid_R
	−58	22	12	2.57767	1,432	Frontal_Inf_Tri_L
	54	−10	−42	2.26472	552	no_label
	50	−28	−4	2.57079	480	Temporal_Mid_R
	58	32	4	2.03234	56	Frontal_Inf_Tri_R
	−8	62	26	2.04844	40	Frontal_Sup_Medial_L
surp_RNNG_TD (uncorrected)	36	−88	−12	3.72838	6,864	Occipital_Inf_R
	−22	−86	−10	2.92842	1,808	Fusiform_L
	−38	−52	−24	2.90338	344	Fusiform_L
	2	−82	2	2.11779	272	Lingual_L
	30	54	26	2.17667	208	Frontal_Mid_2_R
surp_RNNG_LC (uncorrected)	46	−68	48	3.83603	54,024	Angular_R
	50	30	38	3.56834	13,624	Frontal_Mid_2_R
	64	−24	−16	3.56804	7,208	Temporal_Mid_R
	−52	24	34	3.49083	6,264	Frontal_Mid_2_L
	−28	−28	−28	2.74120	4,280	Fusiform_L
	−22	−82	−20	3.11502	3,416	Cerebelum_Crus1_L
	22	−82	−16	3.65646	3,328	Fusiform_R
	22	−16	−34	2.69411	3,264	no_label
	4	8	−16	2.63855	2,128	no_label
	34	52	−4	2.39320	592	Frontal_Mid_2_R
	−40	44	−4	2.31943	544	Frontal_Mid_2_L
	−30	20	60	2.26635	400	Frontal_Mid_2_L
	−66	−12	−10	2.39706	296	Temporal_Mid_L
	66	−4	−2	2.18952	232	Temporal_Sup_R
	−24	64	6	2.04272	176	Frontal_Sup_2_L
	−42	−18	−26	2.11997	152	Temporal_Inf_L
	22	−94	8	1.99267	24	Occipital_Sup_R
dis_RNNG_TD	−34	−74	40	4.21833	6,104	Parietal_Inf_L
	44	−68	44	3.99230	4,488	Angular_R
	−10	−58	14	3.93825	1,280	Precuneus_L
	64	−32	−18	3.64786	872	Temporal_Inf_R
	14	−54	14	3.97503	808	Precuneus_R
dis_RNNG_LC (uncorrected)	−60	−34	40	3.42344	15,392	Parietal_Inf_L
	−50	10	10	3.64759	11,472	Frontal_Inf_Oper_L
	64	−30	46	3.14897	10,424	SupraMarginal_R
	−14	16	60	3.53704	5,760	Frontal_Sup_2_L
	8	−32	−58	2.38765	1,928	no_label
	12	56	28	2.58213	1,240	Frontal_Sup_Medial_R
	−54	−12	−28	2.60976	880	Temporal_Inf_L
	30	24	−18	2.50757	832	Insula_R
	−38	−56	66	2.30140	784	no_label
	−16	−72	66	2.51366	768	no_label
	12	24	56	2.57717	752	Supp_Motor_Area_R
	44	−4	−6	2.36271	664	Insula_R
	−12	52	30	2.32030	336	Frontal_Sup_2_L
	56	−4	52	2.17256	304	Frontal_Mid_2_R
	24	6	−24	2.23977	240	ParaHippocampal_R
	−68	−14	10	2.04049	128	no_label
	60	−6	−38	2.06499	64	Temporal_Inf_R
	56	2	−44	2.18495	48	no_label

*Note*. Thresholded with a false discovery rate = 0.05 and a cluster threshold of 100 voxels. The regions were identified by using AtlasReader (Notter et al., 2019).

The main results are as follows: As for LSTM, although the threshold is uncorrected, the increased activities were confirmed in the right middle temporal pole and the left IFGtriang (Figure 7). Notice that even though the AtlasReader indicates no_label, the increasing activity in the left posterior temporal lobe (PTL) can be observed in Figure 7. Surp_RNNG_TD was associated with the activities in the left fusiform gyrus and the right inferior occipital lobe (using an uncorrected threshold; see Figure 8). Surp_RNNG_LC was associated with activities in the right AG, the right middle temporal lobe and the left middle frontal gyrus (uncorrected; Figure 9). Dis_RNNG_TD was associated with activities in the left parietal lobule, the right AG as well as bilateral precuneus (Figure 10). As for dis_RNNG_LC (uncorrected; Figure 11), the main increased activities were observed in the left parietal lobule and the left IFGoperc.

## DISCUSSION

Our goal for this study was to test not only whether RNNGs better explain human fMRI data than LSTMs, but also whether the left-corner RNNGs outperform the top-down RNNGs. We localized the syntactic composition effects of the left-corner RNNG in certain brain regions, using the information-theoretic metric, such as surprisal, and a metric that measures the syntactic work, that is, distance, to quantify the computational models. Surprisal is assumed to associate with the amount of the cognitive effort in the brain during language comprehension, which has been attested in the previous studies (Bhattasali & Resnik, 2021; Brennan et al., 2020; Henderson et al., 2016; Lopopolo et al., 2017; Willems et al., 2015). In Brennan et al. (2020), the surprisal estimated from LSTM had statistically significant effects for their ROIs such as the left ATL, the left IFG, the left PTL, and the left IPL, against a baseline model. However, our results did not show such effects for the 5-gram model and LSTM across all ROIs. We also adopted another complexity metric, distance, which was tested in Hale et al. (2018) and Brennan et al. (2020) for RNNGs. In Brennan et al. (2020), it was shown that distance calculated from the top-down RNNG had statistically significant effects in the left ATL, the left IFG, and the left PTL, compared to what they called RNNG-comp (a degraded version of RNNGs that does not include the composition function). In our results, dis_RNNG_LC showed statistically significant effects in the IFGoperc, IFGtriang, IFGorb, IPL, AG, STG and sATL, compared to LSTM (Table 6). Our results also found that dis_RNNG_TD improves the model fits to the fMRI data in the IFGoperc, IFGtriang, IPL, AG, and sATL, compared to LSTM. Considering these, we showed in addition to Brennan et al. (2020), that the hierarchical models better explain the fMRI data compared to sequential models.

The results of the whole brain analysis showed that some control predictors such as word rate and word length were involved in regions that are related to the visual processing and the visual word form area such as the fusiform gyrus and the occipital lobe. Since the task was reading sentences segment by segment, the activation of these regions is expected. In terms of sequential models, the activity in the left PTL was associated with LSTM. However, again, the ROI analyses did not show any statistically significant effects for 5-gram < LSTM, and it remains unclear how to interpret the activity in the left PTL for LSTM, at least in this study.

Although the surprisal estimated from the 5-gram model and LSTM did not fit the fMRI data well, the results of our ROI analyses showed that the left-corner RNNG had statistically significant effects in several ROIs, compared to LSTM (Table 4 and Table 6). These results suggest that the syntactic composition with the left corner parser strategy is involved in these regions, and our results align with the previous studies. For example, the surprisal computed from a top-down context-free parser in Henderson et al. (2016) was associated with the activities in the IFG including pars opercularis (BA44), compared to lexical surprisal. There is also a piece of evidence for STG associated with phrase structure grammar. Although they did not use surprisal, in Lopopolo et al. (2021), node count from structures generated by phrase structure grammar was used as a complexity metric, and it showed a significant effect in STG, whereas the dependency grammar (which describes the relationship between a head and its dependent) did not show such an effect in this region, but the middle temporal pole was responsible for this grammar. The result that the node count effect was shown in STG is compatible with our surp_RNNG_LC and dis_RNNG_LC results, but not compatible with the results of surp_RNNG_TD and dis_RNNG_TD. As mentioned above, on the other hand, Henderson et al. (2016) did show the effect in IFG for the surprisal computed from CFGs, but they also reported that they did not observe the effect in STG. These mixed results make it hard to evaluate the effect of STG, though it is considered to be involved in sentence-level comprehension (e.g., Nelson et al., 2017; Pallier et al., 2011).

The regions such as IFGoperc and IPL for dis_RNNG_LC appeared to be important based on our ROI analyses, and the whole brain analyses confirmed the strong activation in these regions. IFG has been attested in the literature in which a simple composition was examined (Friederici, 2017; Maran et al., 2022; Zaccarella & Friederici, 2015). However, several other studies suggest that there is no comprehensive understanding regarding the locus of the composition in the brain (Pylkkänen, 2019, 2020; Pylkkänen & Brennan, 2020). Our results from dis_RNNG_LC partially aligns with Brennan et al.’s (2020) results where the distance computed from top-down RNNGs had a significant effect in IFGoperc as well as in ATL and PTL in their results. Brennan and Pylkkänen (2017) showed that the left-corner CFG was associated with the activation in the left ATL, which our ROI analysis results did not show in the results of dis_RNNG_TD < dis_RNNG_LC (Table 7). However, the sATL effect for dis_RNNG_TD and dis_RNNG_LC was found against LSTM. This might indicate that sATL is involved in composition, but not involved in the effect of the left-corner parsing strategy, compared to the effect of the top-down parsing strategy.

So far, we have discussed the regions that were associated with the left-corner RNNG, but we have not discussed how surprisal or distance computed from the left-corner RNNG modulates in the brain. In previous studies, it has been unclear which brain region is responsible for which component of computational models since the role of the syntactic processing for each study has been observed using different grammars with different complexity metrics: for example, surprisal estimated from part-of-speech (Lopopolo et al., 2017); surprisal computed from CFGs (Henderson et al., 2016); node count from the structures generated by CFGs (Brennan et al., 2012; Brennan & Pylkkänen, 2017; Giglio et al., 2022; Lopopolo et al., 2021); node count from the structures generated by combinatory categorial grammars (Stanojević et al., 2021, 2023); node count from the structures generated by minimalist grammars (Brennan et al., 2016; Li & Hale, 2019); surprisal and distance computed from top-down RNNGs (Brennan et al., 2020). It might be a case where surprisal and the metrics that express the process of the steps (e.g., node count, distance) play roles in designated regions of the brain separately. For example, the steps of structure building might be involved in the PTL (Flick & Pylkkänen, 2020; Matar et al., 2021; Matchin & Hickok, 2020; Murphy et al., 2022), which is compatible with some previous studies (Brennan et al., 2016, 2020; Li & Hale, 2019; Stanojević et al., 2023). Surprisal, on the other hand, might be modulated in more broad regions that have to do with language processing in addition to the process of the steps. This point should be clarified in future work that can test different complexity metrics with different grammars or computational models using the same human data. Related to this discussion, the attempt for identifying the locus of composition has not been converged in the neurobiology of language literature; some studies have argued that a specific part of the Broca’s area is for syntactic composition (or *merge*; Zaccarella et al., 2017; Zaccarella & Friederici, 2015), while others have claimed that the ATL is the locus of semantic composition (Bemis & Pylkkänen, 2011, 2013; Zhang & Pylkkänen, 2015). Another candidate for the syntactic composition is the PTL (Flick & Pylkkänen, 2020; Matar et al., 2021; Matchin & Hickok, 2020; Murphy et al., 2022). Or, the connection between two regions (IFG and PTL) might be a source of syntactic composition (cf. Hardy et al., 2023; Maran et al., 2022; Wu et al., 2019). Although these candidates for syntactic composition are compatible with our results, future work needs to be done.

## CONCLUSION

In this article, we investigated whether hierarchical models like RNNGs better explain human brain activity than sequential models like LSTMs, as well as which parsing strategy is more neurobiologically plausible. As a result, the surprisal metric computed from left-corner RNNGs significantly explained the brain regions including IFGoperc, IFGtriang, IPL, AG, and STG relative to LSTMs, though the surprisal metrics estimated from 5-gram models, LSTMs, and top-down RNNGs did not show any significant effects across eight regions in the ROI analyses. In addition, the distance metric computed from left-corner RNNGs did show significant effects in IFGoperc, IFGtriang, IFGorb, IPL, AG, and STG, relative to the distance metric estimated from top-down RNNGs, but notvice versa. Overall, our results suggest that left-corner RNNGs are the neurobiologically plausible computational model of human language processing, and there are certain brain regions that localize the syntactic composition with the left-corner parsing strategy.

## ACKNOWLEDGMENTS

We thank Haining Cui for fMRI data collection. We are also grateful to two anonymous reviewers for helpful suggestions and comments.

## FUNDING INFORMATION

Yohei Oseki, Japan Society for the Promotion of Science (https://dx.doi.org/10.13039/501100000646), Award ID: JP21H05061. Yohei Oseki, Japan Society for the Promotion of Science (https://dx.doi.org/10.13039/501100000646), Award ID: JP19H05589. Yohei Oseki, Japan Science and Technology Agency (https://dx.doi.org/10.13039/501100002241), Award ID: JPMJPR21C2.

## AUTHOR CONTRIBUTIONS

**Yushi Sugimoto**: Formal analysis: Lead; Investigation: Lead; Methodology: Equal; Software: Lead; Visualization: Lead; Writing – original draft: Lead. **Ryo Yoshida**: Conceptualization: Supporting; Writing – review & editing: Supporting. **Hyeonjeong Jeong**: Methodology: Supporting. **Masatoshi Koizumi**: Project administration: Lead. **Jonathan R. Brennan**: Methodology: Supporting. **Yohei Oseki**: Conceptualization: Lead; Funding acquisition: Lead; Methodology: Supporting; Project administration: Lead; Resources: Lead; Supervision: Lead; Writing – review & editing: Supporting.

## DATA AND CODE AVAILABILITY STATEMENT

The fMRI corpus will be made publicly available in the future. The statistical maps from the whole brain analyses are available on NeuroVault (https://identifiers.org/neurovault.collection:14567). The code for fMRI analyses is available at https://github.com/osekilab/RNNG-fMRI, which is modified from https://github.com/dgd45125/LPPxORCxEN-CN. The code for language models is available at https://github.com/osekilab/RNNG-EyeTrack.

## Supplementary Material


