# Supplementary material for: Localizing Syntactic Composition with Left-Corner Recurrent Neural Network Grammars
Source: Neurobiol Lang (Camb). 2024 Apr 1;5(1):201–24. doi: 10.1162/nol_a_00118 (PMC11025653; doi:10.1162/nol_a_00118)
Supplement: Supplementary file 1 [file nol-5-1-201-s001.pdf]

Supplementary Material for "Localizing Syntactic Composition with Left-Corner Recurrent Neural Network Grammars"

Yushi Sugimoto[1]*, Ryo Yoshida[1], Hyeonjeong Jeong[2], Masatoshi Koizumi[2], Jonathan R. Brennan[3], and Yohei Oseki[1]

[1] University of Tokyo, [2] Tohoku University, [3] University of Michigan

In the following, the beam size differences were tested via `anova()`. The regression models for LSTM and RNNGs were defined as follows.

LSTM_ROI = lmer(ROI ~ LSTM + 5-gram + dx + dy + dz + rx + ry+ rz + word_rate + word_freq + word_length + sentid + sentpos + (1 | subject_number), dataTrim_ROI, REML = FALSE)

RNNG_ROI = lmer(ROI ~ RNNG + LSTM + 5-gram + dx + dy + dz + rx + ry+ rz + word_rate + word_freq + word_length + sentid + sentpos + (1 | subject_number), dataTrim_ROI, REML = FALSE)

Surprisal: Left-corner RNNGs

model comparison	ROI	LogLik	χ^2	p
`anova (LSTM, RNNG_1)`	IFGoperc	-9084.4	14.989	0.0001082 ***
	IFGtriang	-11049	21.378	3.771e-06 ***
	IFGorb	-17914	6.768	0.009281 **
	IPL	-12690	27.109	1.923e-07 ***
	AG	-13405	14.483	0.0001414 ***
	STG	-13834	10.165	0.001432 **
	sATL	-19062	2.5749	0.1086
	mATL	-23917	0.4569	0.4991
`anova (LSTM, RNNG_2)`	IFGoperc	-9084.4	15	0.0001075 ***
	IFGtriang	-11050	20.486	6.008e-06 ***
	IFGorb	-17914	6.1962	0.0128 *
	IPL	-12690	27.352	1.696e-07 ***
	AG	-13405	14.597	0.0001331 ***
	STG	-13834	10.297	0.001333 **
	sATL	-19062	2.5549	0.11
	mATL	-23917	0.3914	0.5315
`anova (LSTM, RNNG_4)`	IFGoperc	-9085.2	13.33	0.0002612 ***
	IFGtriang	-11050	18.427	1.765e-05 ***
	IFGorb	-17915	5.3059	0.02125 *
	IPL	-12691	25.851	3.687e-07 ***
	AG	-13406	13.17	0.0002845 ***
	STG	-13835	8.8692	0.0029 **
	sATL	-19063	1.7879	0.1812
	mATL	-23917	0.2148	0.643
`anova (LSTM, RNNG_6)`	IFGoperc	-9085.3	13.258	0.0002714 ***
	IFGtriang	-11051	18.221	1.967e-05 ***
	IFGorb	-17915	5.3976	0.02016 *
	IPL	-12691	26.057	3.314e-07 ***
	AG	-13406	13.694	0.0002152 ***
	STG	-13835	9.0076	0.002689 **
	sATL	-19062	1.9129	0.1666
	mATL	-23917	0.2879	0.5916
`anova (LSTM, RNNG_8)`	IFGoperc	-9085.4	13	0.0003115 ***
	IFGtriang	-11051	17.877	2.357e-05 ***
	IFGorb	-17915	5.3149	0.02114 *
	IPL	-12691	25.789	3.809e-07 ***
	AG	-13406	13.515	0.0002367 ***
	STG	-13835	8.7552	0.003087 **
	sATL	-19063	1.8502	0.1738
	mATL	-23917	0.2763	0.5991
`anova (LSTM, RNNG_10)`	IFGoperc	-9085.4	13.003	0.0003111 ***
	IFGtriang	-11051	17.864	2.372e-05 ***
	IFGorb	-17915	5.3776	0.0204 *
	IPL	-12691	25.94	3.522e-07 ***
	AG	-13406	13.642	0.0002212 ***
	STG	-13835	8.8212	0.002977 **
	sATL	-19063	1.8739	0.171
	mATL	-23917	0.2966	0.586

Table 1: Results of model comparisons (LSTM < surp_RNNGs_LC): The number followed by RNNG indicates the beam size. For example, RNNG_1 means RNNGs with beam size k =100

Surprisal: Top-down RNNGs

model comparison	ROI	LogLik	χ^2	p
anova (LSTM, RNNG_1)	IFGoperc	-9082.3	19.246	1.149e-05 ***
	IFGtriang	-11054	11.466	0.000709 ***
	IFGorb	-17917	2.0736	0.1499
	IPL	-12694	19.216	1.167e-05 ***
	AG	-13404	17.409	3.014e-05 ***
	STG	-13832	15.568	7.96e-05 ***
	sATL	-19058	12.027	0.0005242 ***
	mATL	-23916	2.3875	0.1223
anova (LSTM, RNNG_2)	IFGoperc	-9085.0	13.874	0.0001954 ***
	IFGtriang	-11056	6.5087	0.01073 *
	IFGorb	-17917	1.487	0.2227
	IPL	-12695	16.564	4.703e-05 ***
	AG	-13408	9.7042	0.001839 **
	STG	-13834	11.721	0.0006179 ***
	sATL	-19060	6.5707	0.01037 *
	mATL	-23916	1.723	0.1893
anova (LSTM, RNNG_4)	IFGoperc	-9091.4	0.9848	0.321
	IFGtriang	-11060	0.0256	0.873
	IFGorb	-17918	0.0969	0.7556
	IPL	-12703	2.2487	0.1337
	AG	-13411	2.5374	0.1112
	STG	-13839	1.4985	0.2209
	sATL	-19063	1.0775	0.2992
	mATL	-23917	0.454	0.5004
anova (LSTM, RNNG_6)	IFGoperc	-9091.5	0.9095	0.3402
	IFGtriang	-11060	1e-04	0.994
	IFGorb	-17918	0.1691	0.681
	IPL	-12703	1.7395	0.1872
	AG	-13412	1.3312	0.2486
	STG	-13838	2.1274	0.1447
	sATL	-19063	1.0054	0.316
	mATL	-23917	0.044	0.8338
anova (LSTM, RNNG_8)	IFGoperc	-9091.3	1.2842	0.2571
	IFGtriang	-11060	0.0612	0.8047
	IFGorb	-17918	0.0064	0.9361
	IPL	-12703	1.5327	0.2157
	AG	-13411	2.9615	0.08527 .
	STG	-13838	3.5706	0.05881 .
	sATL	-19062	2.2315	0.1352
	mATL	-23917	0.4406	0.5068
anova (LSTM, RNNG_10)	IFGoperc	-9090.8	2.2179	0.1364
	IFGtriang	-11060	0.3085	0.5786
	IFGorb	-17918	8 e-04	0.9772
	IPL	-12702	4.0516	0.04413 *
	AG	-13410	3.7982	0.05131 .
	STG	-13837	4.0574	0.04398 *
	sATL	-19062	2.7917	0.09475 .
	mATL	-23917	0.583	0.4452

Table 2: Results of model comparisons (LSTM < surp_RNNGs_TD): The number followed by RNNG indicates the beam size. For example, RNNG_1 means RNNGs with beam size k =100

Distance: Left-corner RNNGs

model comparison	ROI	LogLik	χ^2	p
`anova (LSTM, RNNG_1)`	IFGoperc	-9062.7	58.452	2.083e-14 ***
	IFGtriang	-11039	40.589	1.879e-10 ***
	IFGorb	-17906	23.514	1.24e-06 ***
	IPL	-12682	43.686	3.856e-11 ***
	AG	-13396	-13396	1.151e-08 ***
	STG	-13818	41.917	9.525e-11 ***
	sATL	-19048	31.019	2.555e-08 ***
	mATL	-23915	4.5687	0.03256 *
`anova (LSTM, RNNG_2)`	IFGoperc	-9062.5	58.727	1.811e-14 ***
	IFGtriang	-11039	40.978	1.54e-10 ***
	IFGorb	-17906	22.569	2.027e-06 ***
	IPL	-12681	45.588	1.46e-11 ***
	AG	-13398	28.944	7.452e-08 ***
	STG	-13824	31.384	2.117e-08 ***
	sATL	-19051	24.658	6.845e-07 ***
	mATL	-23915	3.0366	0.0814 .
`anova (LSTM, RNNG_4)`	IFGoperc	-9062.0	59.778	1.062e-14 ***
	IFGtriang	-11039	42.006	9.1e-11 ***
	IFGorb	-17907	21.882	2.899e-06 ***
	IPL	-12682	44.454	2.604e-11 ***
	AG	-13397	29.849	4.671e-08 ***
	STG	-13824	30.705	3.004e-08 ***
	sATL	-19051	25.276	4.969e-07 ***
	mATL	-23915	4.3622	0.03674 *
`anova (LSTM, RNNG_6)`	IFGoperc	-9064.0	55.893	7.654e-14 ***
	IFGtriang	-11040	39.339	3.563e-10 ***
	IFGorb	-17907	20.736	5.273e-06 ***
	IPL	-12681	44.746	2.243e-11 ***
	AG	-13398	28.041	1.188e-07 ***
	STG	-13826	27.119	1.913e-07 ***
	sATL	-19052	22.681	1.913e-06 ***
	mATL	-23915	3.5982	0.05784 .
`anova (LSTM, RNNG_8)`	IFGoperc	-9063.5	56.906	4.572e-14 ***
	IFGtriang	-11040	39.908	2.662e-10 ***
	IFGorb	-17907	20.883	4.883e-06 ***
	IPL	-12681	45.748	1.345e-11 ***
	AG	-13398	28.111	1.145e-07 ***
	STG	-13826	26.824	2.229e-07 ***
	sATL	-19052	23.182	1.473e-06 ***
	mATL	-23915	3.8268	0.05044 .
`anova (LSTM, RNNG_10)`	IFGoperc	-9065.3	53.281	2.891e-13 ***
	IFGtriang	-11041	37.671	8.373e-10 ***
	IFGorb	-17908	19.42	1.049e-05 ***
	IPL	-12682	43.783	3.668e-11 ***
	AG	-13400	25.668	4.054e-07 ***
	STG	-13827	25.079	5.502e-07 ***
	sATL	-19053	21.152	4.243e-06 ***
	mATL	-23915	3.3298	0.06803

Table 3: Results of model comparisons (LSTM < dis_RNNGs_LC): The number followed by RNNG indicates the beam size. For example, RNNG_1 means RNNGs with beam size k =100

Distance: Top-down RNNGs

model comparison	ROI	LogLik	χ^2	p
anova (LSTM, RNNG_1)	IFGoperc	-9081.4	21.015	4.558e-06 ***
	IFGtriang	-11054	10.412	0.001252 **
	IFGorb	-17914	7.3719	0.006625 **
	IPL	-12696	15.746	7.243e-05 ***
	AG	-13404	17.662	2.639e-05 ***
	STG	-13836	5.9249	0.01493 *
	sATL	-19055	16.713	4.349e-05 ***
	mATL	-23915	4.3965	0.03601 *
anova (LSTM, RNNG_2)	IFGoperc	-9079.0	25.743	3.901e-07 ***
	IFGtriang	-11053	12.951	0.0003198 ***
	IFGorb	-17914	8.0722	0.004495 **
	IPL	-12694	18.678	1.548e-05 ***
	AG	-13402	20.182	7.042e-06 ***
	STG	-13834	10.975	0.0009234 ***
	sATL	-19053	21.187	4.165e-06 ***
	mATL	-23915	4.5889	0.03218 *
anova (LSTM, RNNG_4)	IFGoperc	-9079.4	25.022	5.668e-07 ***
	IFGtriang	-11054	12.07	0.0005124 ***
	IFGorb	-17914	6.8511	0.008859 **
	IPL	-12694	19.686	9.126e-06 ***
	AG	-13402	20.865	4.929e-06 ***
	STG	-13834	10.33	0.001309 **
	sATL	-19054	18.927	1.358e-05 ***
	mATL	-23915	3.4689	0.06253 .
anova (LSTM, RNNG_6)	IFGoperc	-9080.7	22.363	2.256e-06 ***
	IFGtriang	-11054	10.418	0.001248 **
	IFGorb	-17915	5.9565	0.01466 *
	IPL	-12693	20.615	5.615e-06 ***
	AG	-13402	20.796	5.108e-06 ***
	STG	-13835	8.088	0.004456 **
	sATL	-19055	16.371	5.207e-05 ***
	mATL	-23915	2.9976	0.08339 .
anova (LSTM, RNNG_8)	IFGoperc	-9080.6	22.563	2.033e-06 ***
	IFGtriang	-11054	10.425	0.001243 **
	IFGorb	-17915	5.7912	0.01611 *
	IPL	-12694	19.74	8.873e-06 ***
	AG	-13402	20.948	4.718e-06 ***
	STG	-13835	8.2117	0.004162 **
	sATL	-19055	16.843	4.059e-05 ***
	mATL	-23915	3.0929	0.07863 .
anova (LSTM, RNNG_10)	IFGoperc	-9082.1	19.688	9.118e-06 ***
	IFGtriang	-11055	8.7038	0.003176 **
	IFGorb	-17915	5.0968	0.02397 *
	IPL	-12695	17.437	2.97e-05 ***
	AG	-13402	19.663	9.239e-06 ***
	STG	-13836	6.948	0.008391 **
	sATL	-19056	15.182	9.762e-05 ***
	mATL	-23916	2.7588	0.09672 .

Table 4: Results of model comparisons (LSTM < dis_RNNGs_TD): The number followed by RNNG indicates the beam size. For example, RNNG_1 means RNNGs with beam size k =100
